# Time-resolved assembly of a nucleoprotein complex between *Shigella flexneri virF* promoter and its transcriptional repressor H-NS

**DOI:** 10.1093/nar/gku1052

**Published:** 2014-11-11

**Authors:** Ulisse Ulissi, Attilio Fabbretti, Marco Sette, Anna Maria Giuliodori, Roberto Spurio

**Affiliations:** 1 Laboratory of Genetics, School of Biosciences and Veterinary Medicine, University of Camerino, Camerino (MC) 62032, Italy; 2 Department of Chemical Sciences and Technologies, University of Rome-Tor Vergata, 00133 Roma, Italy

## Abstract

The *virF* gene of *Shigella*, responsible for triggering the virulence cascade in this pathogenic bacterium, is transcriptionally repressed by the nucleoid-associated protein H-NS. The primary binding sites of H-NS within the promoter region of *virF* have been detected here by footprinting experiments in the presence of H-NS or its monomeric DNA-binding domain (H-NS*ctd*), which displays the same specificity as intact H-NS. Of the 14 short DNA fragments identified, 10 overlap sequences similar to the H-NS binding motif. The ‘fast’, ‘intermediate’ and ‘slow’ H-NS binding events leading to the formation of the nucleoprotein complex responsible for transcription repression have been determined by time-resolved hydroxyl radical footprinting experiments in the presence of full-length H-NS. We demonstrate that this process is completed in ≤1 s and H-NS protections occur simultaneously on site I and site II of the *virF* promoter. Furthermore, all ‘fast’ protections have been identified in regions containing predicted H-NS binding motifs, in agreement with the hypothesis that H-NS nucleoprotein complex assembles from a few nucleation sites containing high-affinity binding sequences. Finally, data are presented showing that the 22-bp fragment corresponding to one of the HNS binding sites deviates from canonical B-DNA structure at three TpA steps.

## INTRODUCTION

Enterobacterial histone-like nucleoid-structuring protein (H-NS) plays a dual role. It contributes to the architectural organization of the nucleoid and acts as a transcriptional repressor on a wide range of genes (∼5% of the total) (1–5), some encoding housekeeping functions, others implicated in cellular responses to environmental changes, including the virulence factors whose expression is triggered by the passage from the external environment to that of the host intestine (6,7).

Different mechanisms, such as (i) promoter occlusion, as postulated for *hns* autorepression (8–10) and (ii) RNA polymerase entrapment in the promoter caused by H-NS-mediated DNA looping (11,12) have been implicated in transcriptional repression by H-NS.

The *Shigella* plasmid-encoded *virF* encodes the activator of the multistep pathogenicity cascade of this enterobacterium and is among the virulence genes subjected to temperature-dependent transcriptional repression by H-NS (13,14). Previous studies have demonstrated that H-NS targets two sites on the *virF* promoter: site I centered at −1 and site II centered at −250, separated by an intrinsic DNA curvature centered around −140 position (14).

In this study, we have used time-resolved chemical probing to investigate the dynamics of the formation of the H-NS-*virF* promoter complex at 20°C, a temperature at which *virF* is subjected to transcriptional repression.

Our results shed some light on the nature of the H-NS nucleation sites of this promoter that is found to contain several DNA consensus sequences where the repressor protein initially binds and from which it spreads to other promoter regions by oligomerization to form the repression complex.

## MATERIALS AND METHODS

### Bacterial strains, plasmids and general procedures

Native H-NS and H-NS*ctd* were obtained upon their hyper-expression from pPLc11 (15) and pEV1_H-NSctd (16) vectors, carrying the entire *hns* sequence and the H-NS sequence from Arg89 to Gln136, respectively, in *Escherichia coli* UT5600 carrying the pcI857 vector encoding the ts λCI repressor. Induction, overexpression and protein purifications were carried out as described (16). The 450-bp DNA fragment corresponding to the *virF* promoter region (14) was generated by polymerase chain reaction (PCR) amplification using pULS55 (17) as template and primers: 5′-GCGAACCTTTATATCT-3′ (BX8) and 5′-CGCTTCCTTTAGCAGC-3′ (FO1) as described (14). In this amplicon, the 37 nucleotides proximal to the FO1 primer belong to the cloning vector. DNA sequencing was performed by the dideoxy chain termination method (18).

### DNase I footprinting experiments

The *virF* promoter fragment was generated by PCR using two primers GEN-348 (5′-GCGAACCTTTATATCT-3′) and GEN-349 (5′-CGCTTCCTTTAGCAGC-3′). Labeling of one DNA end was achieved by introducing [γ-^32^P] ATP (3000 Ci/mmol) at the 5′ end of the molecule, using phage T4 polynucleotide kinase. The DNA fragment containing the *virF* promoter was then incubated for 10 min at 20°C with increasing amounts of either H-NS or H-NS*ctd* in 70 μl of 20 mM Tris-HCl (pH 7.7) buffer containing 8% glycerol, 85 mM NH_4_Cl, 15 mM KCl, 1.5 mM Mg-acetate and 0.5 mM DTT. After addition of DNase I (0.75 μg) and 30 s incubation, the reaction was stopped by addition of 70 μl of 100 mM ethylenediaminetetraacetic acid (EDTA) and placing the samples on ice. After phenol-chloroform extraction, the samples were precipitated with three volumes of ethanol in the presence of 1 M NH_4_ acetate pH 7.4 and 1 μg tRNA carrier. Samples were then resuspended in 10 μl of formamide blue buffer (90% v/v formamide, 10 mM EDTA pH 8.0, 0.025% w/v bromophenol blue, 0.025% w/v xylene cyanol) and loaded on 7% sequencing gel.

### Hydroxyl radical footprinting experiments

The Fe(II)-EDTA–induced DNA cleavage and the subsequent quenching by addition of absolute ethanol and 100 mM Na acetate (pH 5.4) were carried out at 20°C, essentially as described (19,20). The footprinting experiments were carried out using DNA fragments (100 ng/sample) obtained as described above, in the presence of the H-NS concentrations and for the times indicated in each experiment.

### Time-resolved hydroxyl radical footprinting experiments

Fast mixings were performed in a BioLogic (Grenoble, France) SFM-400 quenched-flow apparatus containing four syringes and two variable delay lines. The first two syringes contained the components to be mixed, the third contained the chemical modification reagent and the fourth a quench solution to stop the reaction. The incubation time of the various components and the modification time can be varied by changing the delay lines and/or the flow parameters. For the hydroxyl radical footprinting experiment, two syringes filled with the assay buffer (20 mM sodium cacodylate, 0.2 M NaCl) were used to drive the DNA-H_2_O_2_ (0.45%) and H-NS samples contained in the ‘sample loops’ (30 μl each) into a ‘reaction loop’, where they were mixed at the concentrations of 5.7 nM and 1.3 μM for the DNA and the protein, respectively. After the indicated pause intervals, the protein-DNA mixture was pushed into contact with the Fe-EDTA solution (15 mM), supplied by the third drive syringe at the end of the reaction loop. The cleavage reaction was quenched completely by 470 μl of 96% ethanol and 100 mM Na-acetate (pH 5.4), supplied by the fourth syringe. After quenching and ethanol precipitation, each sample was resuspended in 200 μl of H_2_O, subjected to phenol-chloroform extraction, precipitated again with three volumes of ethanol in the presence of 1 M NH_4_ acetate (pH 7.4) and 1 μg carrier tRNA. Each sample was then resuspended in 40 μl of H_2_O and 5 μl were mixed with Taq reaction buffer (67 mM Tris-HCl pH 8.8; 16.6 mM (NH_4_)_2_SO_4_; 0.1% (v/v) Tween-20); 3.0 mM MgCl_2_; 100 μM each of the four dNTPs; 0.5 U of Taq DNA polymerase (Polymed) and 4 pmoles of 5′-^32^P-labeled primer, to a final reaction volume of 10 μl. The sequences of these primers were: BX8 (from +105 to +90): 5′ GCGAACCTTTATATCT-3′; FO1 (from −345 to −330): 5′-CGCTTCCTTTAGCAGC-3′; Forw-site I (from −188 to −169): 5′ CAGATATTGCTAAGAAAAGT-3′; Rev-site II (from −90 to −106): 5′ GAGCTTATGCAGCTTCT-3′). After 30 cycles of linear PCR (denaturation 30 s at 95°C, annealing 30 s at 50°C, except for primer BX8 annealed at 48°C, extension 30 s at 72°C) the extension products were separated on 7% acrylamide DNA sequencing gel. To rule out the possibility that H_2_O_2_ may produce artifacts due to oxidative damage of the protein, a control experiment was also conducted incubating H-NS with H_2_O_2_ in syringe 1, DNA alone in syringe 2 and Fe-EDTA solution in syringe 3. The resulting pattern of protection of DNA sites on *virF* promoter was comparable to the one obtained with H_2_O_2_ present in syringe 2 (data not shown). The control samples indicated as zero millisecond represent the mixture DNA-H_2_O_2_ without protein.

### Quantitative gel analysis

Digital images of gel autoradiograms were acquired using a Molecular Dynamics Phosphor Imager (Molecular Imager FX). The intensity of each band, for at least two gel autoradiograms, was quantitatively analyzed on both strands with Image Quant software (Molecular Dynamics) and the resulting values were averaged. The time-dependent change in the intensity of each band corresponding to DNA sites protected by H-NS was fit to single exponential equations.

### Nuclear magnetic resonance (NMR) spectroscopy

NMR spectra of the 22-bp DNA fragment were collected at 900 MHz in both H_2_O and D_2_O as previously described (16). Data were processed using NMRPipe (21) and the full proton spectrum was assigned using Sparky (T.D. Goddard and D.G. Kneller, SPARKY 3, University of California, San Francisco).

## RESULTS

### Comparison of H-NS and H-NSctd binding sites within the virF promoter region

**Figure 1.**
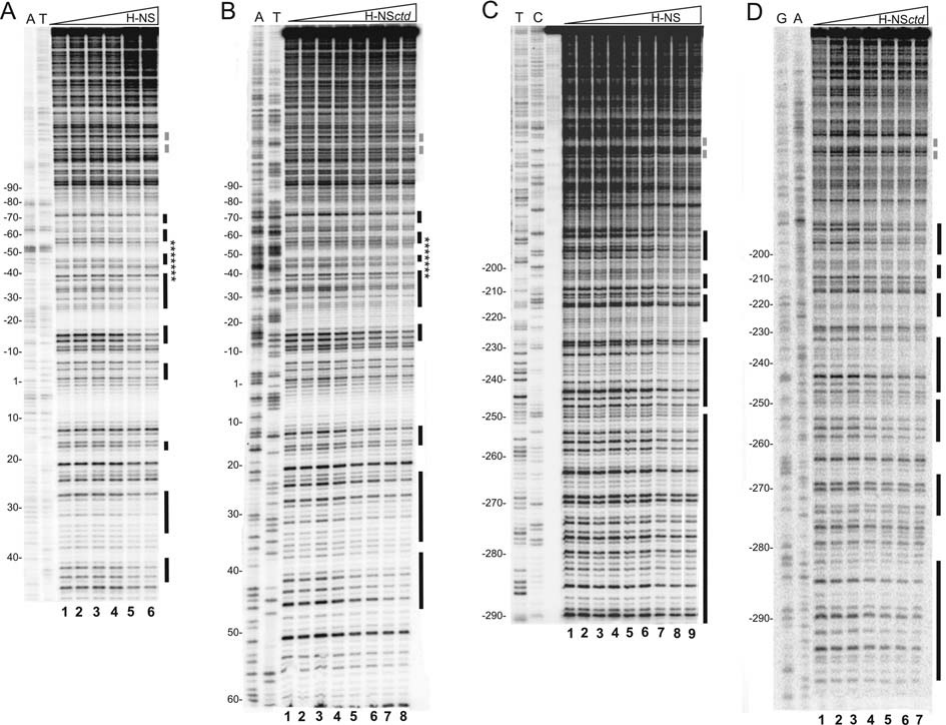
DNase I footprinting of the *virF* promoter with H-NS and H-NS*ctd* at equilibrium. A 450 bp DNA fragment containing *virF* promoter labeled on the non-coding (A and B) and coding (C and D) strands was incubated with DNase I and H-NS or H-NS*ctd* as described in Materials and Methods. The concentration of the proteins was: (A) 0, 0, 23, 46, 114, 230 nM of H-NS dimers from lanes 1 to 6, respectively; (B) 0, 0, 13, 26, 52, 104, 157, 208 μM of H-NS*ctd* monomers from lanes 1 to 8, respectively; (C) 0, 0, 23, 46, 69, 92, 114, 138, 230 nM of H-NS dimers from lanes 1 to 9, respectively; the lane preceding the samples subjected to enzymatic digestion contains the undigested DNA fragment. (D) 0, 13, 26, 52, 104, 157, 208 μM of H-NS*ctd* monomers from lanes 1 to 7, respectively. T, C, A and G lanes represent sequencing reactions performed using primer GEN-348 for A and B and primer GEN-349 for B and D. Black bars indicate the protected sites; stars indicate the sequence of the 22mer fragment analyzed by NMR spectroscopy; broken gray lines indicate the center of the main curvature found in the *virF* promoter region (41). Numbering is given according to the +1 transcription start site.

The H-NS binding sites on the *virF* promoter had been previously identified by DNase I footprinting experiments. However, due to the large dimension of the DNA fragment and to the extensive oligomerization of H-NS, the quality of the footprints was not adequate to distinguish the fine details of the H-NS-DNA interaction (14). Thus, to determine more precisely extension and boundaries of the H-NS target sites at equilibrium, we used the 5′-^32^P-labeled 450 bp DNA fragment obtained by amplification of the +104 and −345 region of *virF.* This portion of the promoter region was subjected to a DNase I footprinting assay in the presence of increasing amounts of either intact H-NS (Figure 1A and C) or of its C-terminal domain (HNS*ctd*) (Figure 1B and D). We tested this domain because it is capable of sequence-selective DNA binding but lacks the oligomerization activity (22,16). Therefore, it offers a unique opportunity for studying the basal characteristics of the H-NS/DNA interaction without interference arising from the formation of large H-NS-DNA complexes.

The footprinting experiments carried out with full-length H-NS yielded protection patterns (indicated by vertical bars in Figure 1 and highlighted in different colors in Supplementary Figure S1) in overall good agreement with those previously obtained (14); site I spanned approximately from +48 to −90 and included both −35 and −10 elements of the promoter while site II spanned from −180 to −290. H-NS protects from DNase I cleavage a total of 14 well-defined DNA regions in site I and site II. As seen from Figure 1A, eight H-NS-protected regions are visible within site I: the first extends from position +43 to +40, the second from position +34 to +27, the third from +17 to +15, the fourth from −14 to −17, the fifth from −27 to −39, the sixth from −45 to −48, the seventh from −57 to −62 and the eighth from −68 to −72. In site II (Figure 1C) the protections are more extended and involve six regions that span from −287 to −272, from −269 to −250, from −247 to –228, from −223 to −215, from −208 to −201 and between −195 and −180, respectively. The same experiments carried out with H-NS*ctd* (Figure 1B and D) resulted in two areas with protection patterns essentially identical to those observed with H-NS. The first area extends from position −10 to −70 within site I while the second is located within site II and spans from position −247 to −180 (with the exception of bases from −233 to −228 and from −208 to −206). However, due to its lack of oligomerization capacity, H-NS*ctd* displays a much lower affinity for these sites compared to full-length H-NS so that the protections appear only at micromolar concentrations of H-NS*ctd* (instead of nanomolar as in the case of H-NS). Also, H-NS*ctd* shields positions from −290 to −283, from −274 to −267, from −259 to −250, from +13 to +17, from +23 to +37 and from +40 to +43. Notably, in contrast to intact H-NS, H-NS*ctd* does not protect positions from −266 to −260, and from −282 to −274.

**Figure 2.**
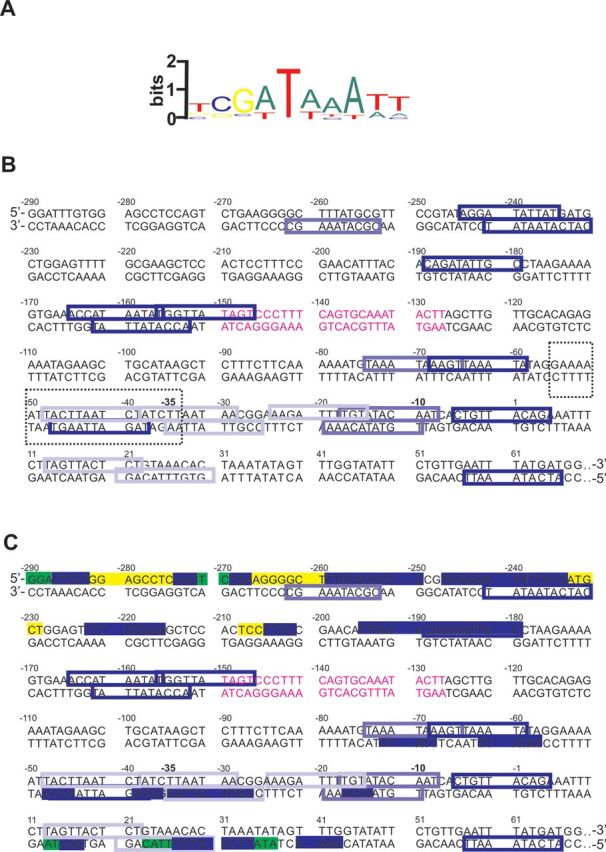
H-NS and H-NS*ctd* binding sites within the sequence of the *virF* promoter region. (A) Logo representation of the H-NS binding motif (22). (B) The relevant portion of the *virF* promoter region is shown. The colored boxes correspond to the H-NS binding motif-like sequences predicted by *in silico* analysis: blue indicates the sequences with at least 4/6 bases identical to the core sequence of the H-NS binding motif and less frequent bases in the other positions; electric blue indicates the sequences with at least 4/6 bases identical to the core sequence of the H-NS binding motif and underrepresented bases in the other positions; powder blue indicates the sequences with 3/6 bases identical to the core sequence of the H-NS binding motif and less frequent bases in the other positions. The dashed line box contains the sequence of the 22mer fragment analyzed by NMR spectroscopy. The sequence shown in purple indicates the center of the main curvature found in the *virF* promoter region (41). (C) Protections from DNase I digestion found with both H-NS and H-NS*ctd* (blue), H-NS only (yellow) and H-NS*ctd* only (green) are highlighted within the sequence of the *virF* promoter region described above.

In 2007, Lang *et al.* have derived a DNA binding motif (Figure 2A) by comparing various H-NS binding site identified mainly by footprinting experiments. Thus, by means of a software that allows identification of a sequence motif in 5′–3′ direction on both DNA strands (23), we used this motif to predict *in silico* the potential H-NS binding sites present in the *virF* promoter. The resulting predicted sites, that are all imperfect fits to the H-NS motif, are shown in Figure 2B. A subsequent inspection of the DNA regions protected by H-NS and H-NS*ctd* within the *virF* promoter (Figure 2C) revealed that 6 out of the 14 regions targeted by H-NS contain a H-NS binding motif-like sequence, 4 contain the binding motif-like sequence in the complementary strand and 3 are T- or AT-rich. As for H-NS*ctd*, this domain targets preferentially short regions that either contain H-NS binding motif-like sequences in one of the two strands (10 out of 15 protected sites) or are T- or AT-rich (4 out of 15 protected sites). Notably, the region spanning from position −282 to −263, which does not resemble the H-NS binding motif and is GC-rich, is protected prevalently by H-NS but not by H-NS*ctd*. Since the latter lacks the oligomerization activity, the short sites protected by H-NS*ctd* could represent the initial H-NS targets in building up the *virF* promoter-H-NS transcription repression complex.

### The primary DNA targets of H-NS are sequence- and structure-specific

Since it had been suggested that H-NS recognizes its sequence consensus target by virtue of a deviation from the canonical B-DNA structure due to the presence of TpA steps (22,16), a DNA region overlapping two targets recognized by both H-NS and H-NS*ctd* was analyzed for possible peculiar structural features which might lead to preferential HNS binding. Thus, a 22-bp DNA fragment corresponding to the sequence comprised between −34 and −55 of the *virF* promoter was analyzed by NMR spectroscopy. This fragment was chosen because it contains three pairs of TpA steps and encompasses two of the aforementioned predicted H-NS binding sites, one of which with high fit to the H-NS binding motif.

**Figure 3.**
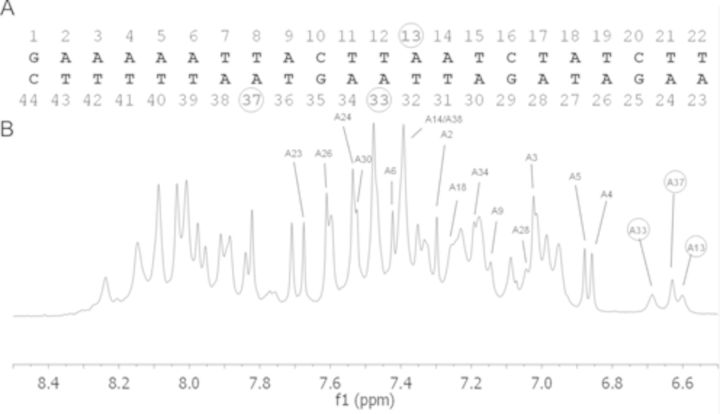
NMR spectrum of a 22-bp DNA fragment containing H-NS targets. (A) Sequence of the 22-bp DNA fragment used for NMR spectroscopy corresponding to the *virF* promoter from −34 to −55. The nucleotides are numbered from 5′ to 3′ on both strands. (B) Proton NMR spectrum of the DNA 22-mer; the three adenines giving upfield-shifted H2 resonances are circled.

Upon resonance assignment (16) of this fragment we could detect non-standard spectroscopic properties, such as an altered chemical shift and line broadening for both H2 protons of adenines A13, A33 and A37. Adenine A13 and A33 are present on opposite filaments within the consensus sequence, while A37 belongs to a different TpA step, located just outside the consensus sequence (Figure 3). These findings indicate that the H-bond pairings of these adenines with their complementary thymines are less stable than normal, a finding that suggests the existence of a local distortion in the corresponding positions of the fragment. This structural perturbation, which involves two TpA steps out of three pairs present in this DNA fragment, is likely stronger than that observed in the *hns* promoter where only one adenine shows altered spectroscopic properties (16).

The occurrence of altered base pairing in this fragment is compatible with its very low melting temperature (data not shown), a property that might be relevant in determining the temperature-dependence of H-NS binding and transcriptional inhibition of the *virF* promoter (14).

### Time-resolved DNA footprinting experiments

Following the identification of the H-NS primary binding sites through the use of the monomeric DNA-binding domain H-NS*ctd*, we sought to test the hypothesis (22) that at least some of the short sequences targeted by this domain might represent the nucleation sites from which H-NS oligomerization could start the process of building a transcriptionally silenced *virF* promoter. Thus, the dynamics of the assembly of a ‘repression complex’ was studied by a series of semi-quantitative time-resolved DNA footprinting assays monitoring H-NS-dependent protection from DNase I and hydroxyl radical cleavages. For these experiments we used the 450-bp DNA fragment derived from the *virF* promoter and a fixed amount of H-NS at which all H-NS binding sites are saturated. Both DNA strands were analyzed in a time scale ranging from 45 to 1200 ms at a constant temperature of 20°C, as described under Materials and Methods.

**Figure 4.**
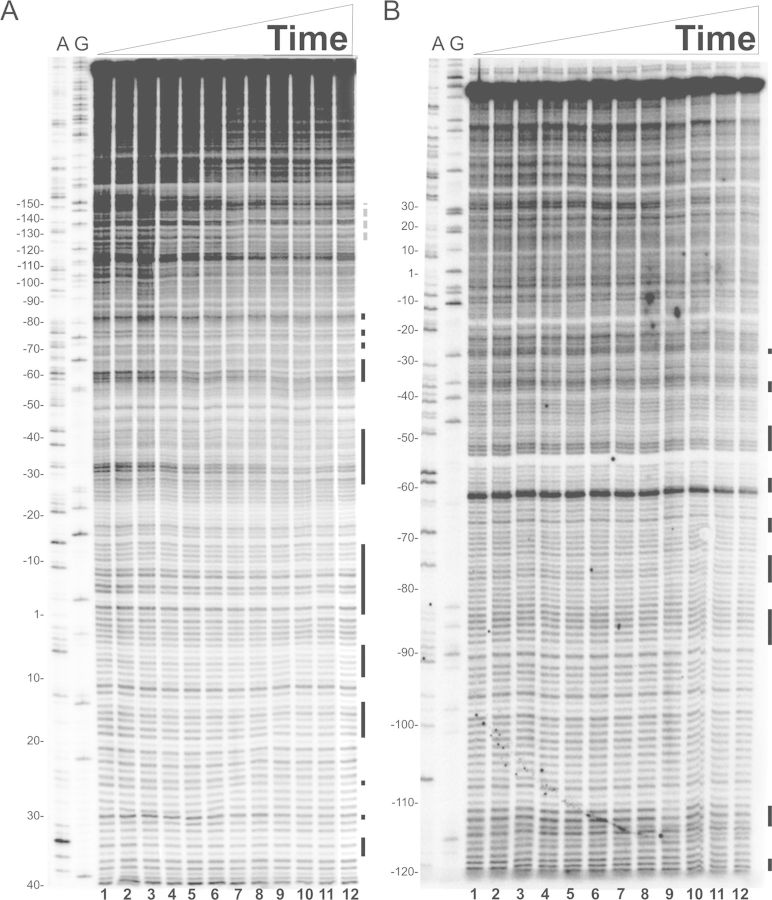
Time-resolved hydroxyl radical H-NS footprinting experiments on site I. Time-resolved hydroxyl radical H-NS footprinting analysis of the coding (A) and non-coding (B) strands corresponding to binding site I of the *virF* promoter. The times elapsed from the mixing of the DNA with H-NS were (A) lanes from 1 to 12: 0, 0, 45, 47, 55, 65, 75, 95, 150, 300, 600 and 1200 ms; (B) lanes from 1 to 12: 0, 0, 45, 45, 49, 55, 65, 75, 95, 150, 600 and 1200 ms. A and G lanes represent sequencing reactions performed with the same primer used for the primer extension analysis. Black bars represent the regions displaying the strongest protections in site I; broken gray line indicates the center of the main curvature. Numbering is given according to the +1 transcription start site. Further details are given in Materials and Methods.

The time-resolved footprinting experiments were initially carried out by a quantitative analysis of the sites protected from DNase I cleavage essentially as described (24). However, DNase I proved not to yield a very accurate pattern of time-resolved DNA cleavage within the very short (milliseconds) range of incubation times. Thus, we decided to analyze the footprints produced by cleavage with hydroxyl radicals generated by the Fenton–Haber–Weiss reaction (25,26) from a free Fe(II)-EDTA complex, (Fe(II) + H_2_O_2_→Fe(III) + OH^**.**^ + OH^-^). Since the cleavage occurs with no sequence-dependence, all backbone positions may be monitored for contact with protein, thereby probing their *in situ* accessibility and providing details about the DNA-protein complex with an accuracy that is not obtainable using the DNase I footprinting technique (27). The results produced by this more direct method indicate a clear, time-dependent protection of the *virF* promoter by H-NS. Since the H-NS protection patterns from hydroxyl radical cleavage at 1200 ms (Figures 4 and 6) and DNase I cleavage at equilibrium (Figure 1) are similar, we conclude that the formation of the H-NS-*virF* promoter complex is completed within ≤1 s.

**Figure 5.**
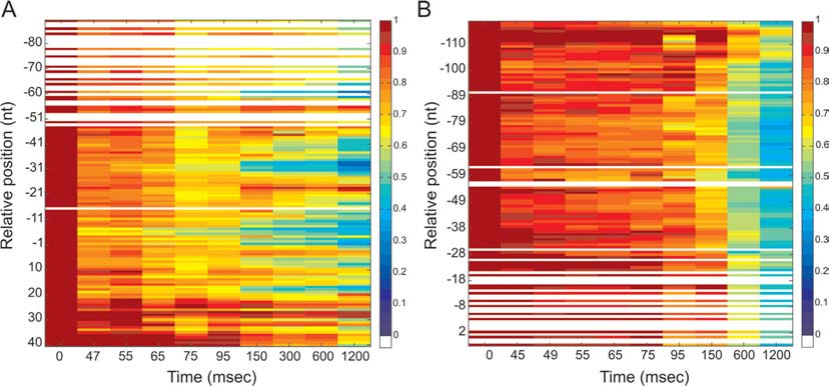
Quantification of H-NS protections detected by footprinting analysis on site I of the *virF* promoter. The normalized intensity of the electrophoretically resolved bands of gels shown in Figure 4A and B is represented by boxes of different colors in panels A and B, respectively. Data were plotted in a linear scale as a function of the time elapsed since the mixing of the DNA with H-NS. A change in color indicates a change in band intensity due to H-NS binding, according to the color key shown in the panels. In brief, dark red corresponds to the intensity measured at *t* = 0 and taken as 100%, red corresponds to a reduction in intensity up to 20%, orange to a reduction between 20–33%, yellow between 33% and 38%, green between 38% and 50%, light blue between 50% and 63%, blue between 63% and 83%, indigo between 83% and 100%. White lines indicate absent data or data of poor quality. Numbering is given according to the +1 transcription start site. The intensity of each band was obtained as described in the text and in Materials and Methods.

**Figure 6.**
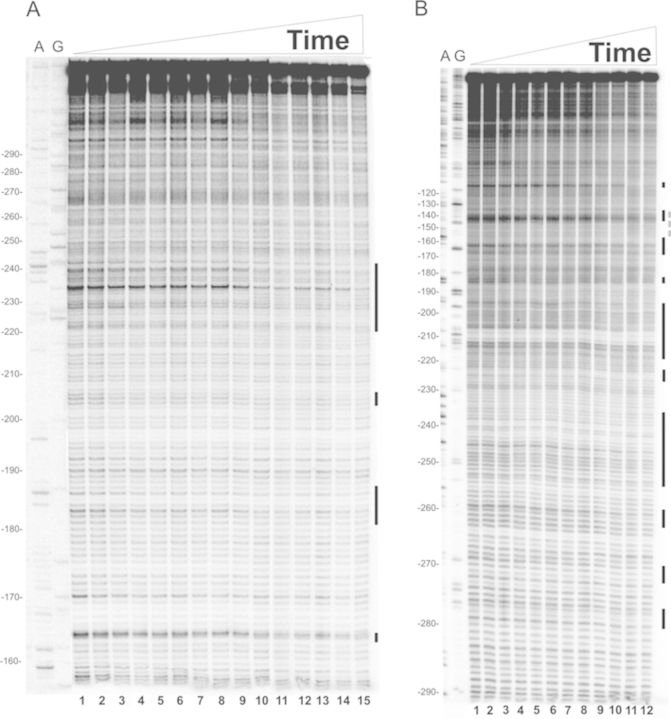
Time-resolved hydroxyl radical footprinting experiments on site II. Time-resolved hydroxyl radical H-NS footprinting analysis of the coding (A) and non-coding (B) strands corresponding to binding site II of the *virF* promoter. The times elapsed from the mixing of the DNA with H-NS were (A) lanes from 1 to 15: 0, 0, 45, 45, 47, 52, 55, 65, 75, 110, 150, 300, 600, 1200 and 3000 ms; (B) lanes from 1 to 12: 0, 0, 45, 47, 55, 65, 75, 95, 150, 300, 600 and 1200 ms. A and G lanes represent sequencing reactions performed with the same primer used for the primer extension analysis. Black bars represent the regions displaying the strongest protections in site II; broken gray line indicates the center of the main curvature. Numbering are given according to the +1 transcription start site. Further details are given in Materials and Methods.

The main H-NS-protected sites found on both strands are contained within three regions in site I (Figure 4A and B), spanning from +20 to −15, −30 to −45 and −60 to −90, and three regions in site II (Figure 6A and B) extending from −200 to −210, −180 to −190 and −220 to −260.

**Figure 7.**
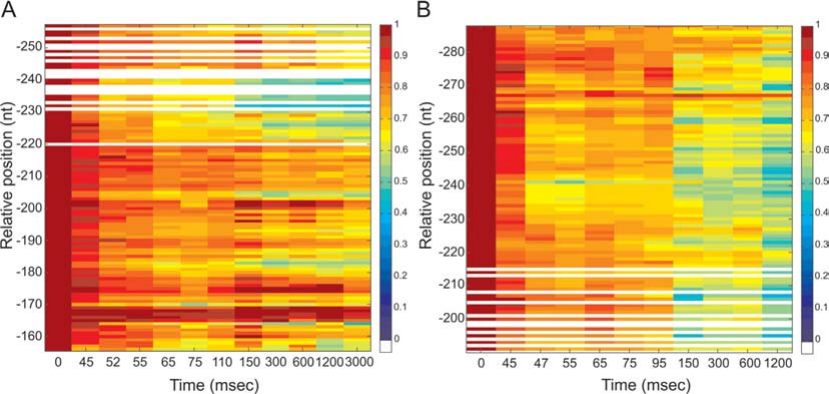
Quantification of H-NS protections detected by footprinting analysis on site II of the *virF* promoter. The normalized intensity of the electrophoretically resolved bands of gels shown in Figure 6A and B is represented by boxes of different colors in panels A and B, respectively. Data were plotted in a linear scale as a function of the time elapsed since the mixing of the DNA with H-NS as described in the legend of Figure 5. Further details are given in the text.

The kinetics of H-NS binding to *virF* promoter, as deduced from these time-resolved footprinting experiments, were derived as follow. The intensity of each electrophoretically resolved band was quantitatively determined by densitometric analysis as described in Materials and Methods. Only the bands whose intensity was above the sensitivity of the instrument were taken into account. Subsequently, each band intensity was normalized by dividing its value by the intensity of the same band measured at time = 0 (in the absence of H-NS). The resulting values were plotted for each time point as colored boxes (Figures 5 and 7). Each color was given according to the extent of shielding from Fe-EDTA cleavage due to H-NS binding. Thus, red indicates regions not protected by H-NS, orange indicates regions scarcely protected, yellow indicates a protection between 33% and 38%, green a protection between 38% and 50%, cyano between 50% and 63% and blue between 63% and 100%. Hence, each analyzed position in the *virF* promoter corresponds to a box whose color may vary over time. Since the timing of yellow, green, cyano or blue appearance in the red/orange background relates to the speed at which the interaction between H-NS and *virF* promoter takes place, this type of data plotting produces a colored map of ‘fast’, ‘intermediate’ and ‘slow’ sites that can be easily interpreted.

Upon inspection of these plots, it is evident that the H-NS protections occur more rapidly in a few defined regions of both sites I and II. In site I, the first H-NS binding takes place in the region located between −5 and −7 in the coding strand (Figure 5A). This fast protection, which occurs within 50 ms, is immediately followed by two additional binding events in upstream regions (i.e. −31 to −33 and from −59 to −61). After these initial events, new ‘intermediate’ binding sites appear within 95–110 ms in both the coding strand (between +19 and +17 and around position 7) and the non-coding strand (around positions −70, −77). During the next 50 ms, H-NS engages both upstream and downstream portions of all targets, covering the surrounding nucleotides. This expansion continues in the next millisecond until it eventually leads to an extended protection of site I by H-NS.

Concerning site II, H-NS cover several targets located approximately from −150 to −280 (Figure 6A and B). The kinetics of protection observed within this site (Figure 7A and B) suggests that H-NS binds faster (within the first 50 ms) and with high affinity to a centrally located area spanning from −225 to −243 on both DNA strands. Another ‘fast’ protection, which concerns nts −182 and −183 in the coding strand, rapidly spreads on the adjacent nucleotides in the subsequent 50 ms. Within the same time window, H-NS also binds two new regions, one located between −200 and −210 and the other between −220 and −230. Finally, when time approaches 150 ms, the ‘slow’ binding sites appear either in new portions of *virF* promoter, such as between −193 and −195, and around positions −250, −260, −270 and −280, or in the complementary strand of previously bound sites.

Inspection of the DNA sequences corresponding to the ‘fast’ H-NS binding targets reveals that all of them show a good correspondence to the sequences targeted by H-NS*ctd* (Figure 2) with the exception of the region comprised between –1 and –11 that was not identified by DNase I footprinting analysis. Furthermore, all ‘intermediate’ binding sites correspond to regions protected by H-NS*ctd* or H-NS in the DNase I experiments and the majority of the ‘slow’ binding sites match the regions protected by H-NS but not by H-NS*ctd*. Therefore, we can conclude from our results that most of the H-NS*ctd*-target sequences indeed represent the nucleation sites where H-NS binds during the initial stages of building the transcription-repression complex on the *virF* promoter.

## DISCUSSION

H-NS, one of the most abundant nucleoid-associated proteins in enterobacteria, is involved in the architectural organization of the chromosome as well as in the transcriptional regulation of a large number of genes (28–32). For many years H-NS has been considered a DNA-binding protein without any sequence specificity and its ability to recognize selective regions of the chromosome was attributed to its capacity to bind preferentially and stabilize intrinsically curved DNA and/or actively induce bending (33–37). However, the finding that the monomeric C-terminal domain of H-NS (HNS*ctd*) displays the same binding selectivity as the native protein and yields well-defined footprints unlike the intact, oligomerizing H-NS (16,38) prompted a careful comparison of the preferred H-NS targets in different H-NS-sensitive promoters. This analysis led to the identification of short consensus sequences selectively recognized by H-NS; in turn, these sequences were suggested to represent high-affinity nucleation sites where the protein binds before building more complex nucleoprotein structures by spreading, through its oligomerization, along lower affinity secondary sites (22,39). The validity of this hypothesis has been checked in the present study analyzing the dynamics of the assembly by H-NS of a transcription repression nucleoprotein complex.

For this study we have used time-resolved DNA footprinting, in the 45–1200 ms range, to study binding of H-NS to the *Shigella virF* promoter. V*irF* was selected because the mechanism of its repression is particularly intriguing (14,40,41) because H-NS yields very extended and complex footprints on its promoter (14) and, last but not least, for the importance of this gene, whose activation triggers the pathogenicity cascade in *Shigella* (7,42,43). Concerning the binding of H-NS, previous studies have demonstrated that H-NS binds to two very extended regions of the *virF* promoter, designated as site I and site II, which are separated by an intrinsic, temperature-sensitive curvature that is not involved in the formation of the protein-DNA complex (14,41). However, these earlier studies had not clarified what drives H-NS to its targets and the dynamics of the formation of the transcription repression complex.

Thus, in the present study through the comparison of H-NS and its monomeric isolated H-NS*ctd*, we have identified 13 short DNA sequences preferentially recognized by both H-NS*ctd* and H-NS in site I and site II. Among these targets, two in site I and two in site II totally overlap sequences matching a medium fit H-NS consensus sequence (at least 4/6 bases identical to the core sequence of the H-NS binding motif), two located in site I overlap a sequence matching a low fit consensus sequence (at least 3/6 bases identical to the core sequence of the H-NS binding motif), four overlap sequences complementary to predicted H-NS binding motifs and three do not mach any motif but fall into T-rich regions. Therefore, our results fully support the hypothesis that H-NS*ctd* could be successfully used to identify preferred H-NS targets, thus avoiding possible interference due H-NS oligomerization activity.

**Figure 8.**
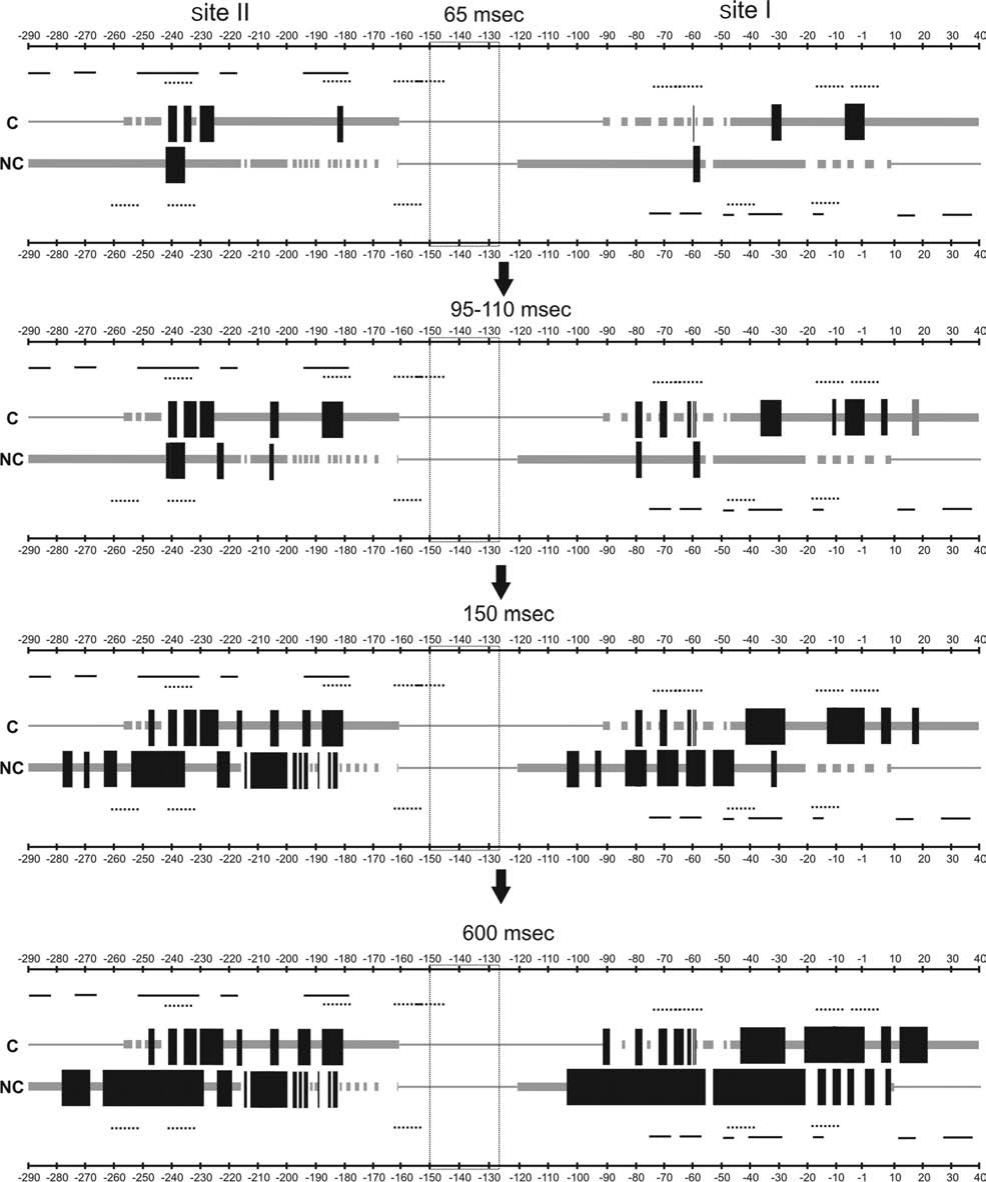
Schematic representation of the time-resolved H-NS binding events on the *virF* promoter region. The coding (C) and non-coding (NC) DNA strands of the v*irF* promoter are depicted as parallel lines, thicker in the portions representing the regions analyzed in this work. The black blocks placed on the strands indicate H-NS protections from Fe-EDTA cleavage of the corresponding phosphodiester bond. Only H-NS binding events which resulted in >25% Fe-EDTA shielding after 65, 95–110, 150 and 600 ms are shown. The H-NS protection pattern is in scale and the numbering is given according to the +1 transcription start site. Gaps interrupting both lines and blocks correspond to regions in which the effect of H-NS binding could not be clearly determined. The location of the main curvature is indicated by a box placed between site I and site II. The lines positioned above the C-strand and below the NC-strand indicate the predicted H-NS binding motifs (dotted lines) and the H-NS*ctd* binding sites detected by DNase I footprinting analysis (solid lines). Only the sequences with at least 4/6 bases identical to the core sequence of the H-NS binding motif are shown. See the text for further explanation.

The analysis of the assembly dynamics of the H-NS-*virF* promoter complex, as it emerges from time-resolved hydroxyl radical footprinting experiments, reveals that: (i) the entire process is completed in ≤1 s at 20°C; (ii) H-NS occupies simultaneously site I and site II (Figures 5 and 7), in agreement with its comparable affinity for the two sites (14) and (iii) H-NS binds its target sites with a precise distribution in terms of space and time, as summarized in Figure 8. In this schematic picture, which shows the dynamics of complex formation in four sequential panels, the two DNA strands of the ***v**irF* promoter are depicted as parallel lines, thicker in the portions indicating the analyzed regions and the protections from Fe-EDTA cleavage occurring at 65, 95–110, 150 and 600 ms after mixing H-NS and DNA.

As can be deduced from the scheme, the protections detected on the coding strand are overall in good agreement with those found on the non-coding strand. More importantly, several protections display an ∼10 base-pair periodic pattern thus suggesting that H-NS prefers to spread locally on the same DNA side occupied during the nucleation step. As for the kinetics of H-NS binding, we found that in building the transcriptional repression nucleoprotein complex, H-NS binds first between −1 and −10 in site I and between −230 and −240 in site II, immediately after covers positions between −30 and −40, −59 and −61 and −180 and −190, and finally involves positions from −200 to −210, from −220 to −230 and around positions +18, +7, −60, −70, −80, before spreading to neighboring nucleotides both upstream and downstream the targets. Notably, fast binding sites are often surrounded by intermediate or slow binding sites, a distribution which clearly describes the time-dependent formation of the complex on the DNA molecule. Therefore, our data fully supports the hypothesis that H-NS assembles the nucleoprotein complex on *virF* promoter starting from a few nucleation sites and subsequently spreads upstream and downstream. Overall, the timing of the interactions deduced from Figure 8, seems to indicate a faster occupancy of the regions pointing toward the center of curvature from site I and site II.

Three out of the five fast protections overlap completely or partially sequences that match a medium fit H-NS binding motif (regions from −230 to −240, −180 to −190, −1 to −10), one overlaps a sequence matching a low fit consensus sequence (from −30 to −35) and one a sequence complementary to a predicted H-NS binding motif (region around −60). Furthermore, two out of the nine intermediate protections fall into a sequence that matches a predicted H-NS binding motif and seven are found in AT-rich regions. On the contrary, slow protections can be found even in regions (i.e. around positions −100 and between −270 and −280) highly divergent from the H-NS consensus sequence. Therefore, the presence of a H-NS binding motif-like sequence seems to favor a rapid H-NS binding. However, not all sequences matching a H-NS binding motif coincide with fast/intermediate H-NS binding sites and, perhaps more important, some sequences that are expected to be recognized by H-NS were not protected by the protein in the footprinting experiments (for example, regions from −250 to −260 and from −40 to −50). This observation suggests that DNA recognition by H-NS is not exclusively sequence based but is also likely influenced by other parameters, such as, for instance, subtle local geometric facets acquired by the target as a function of the nature of the neighboring sequences. A similar conclusion was reached in a study based on protein binding microarray, where the structural information contained within AT-rich minor groove was identified as an essential element for nucleation of H-NS on DNA (44). In this connection, our structural analysis based on the NMR spectra of a DNA portion recognized by both H-NS and H-NS*ctd* within the *virF* promoter, shows that the structure of this fragment deviates somewhat from the classical B-DNA structure; two adenines (A13 and A33) constituting TpA steps on opposite filaments and a third adenine (A37) belonging to a different TpA step, just outside the H-NS consensus sequence, form unstable pairings with their complementary thymines.A similar situation, but restricted to just one adenine, has been observed also in the main binding site of H-NS on the promoter of its own gene *hns* (16).

Thus, since even small distortions of base pairing can lead to modifications of the regular B-DNA structure altering the local shape (e.g. minor groove geometry) and biophysical properties (e.g. electrostatical potential, flexibility) of DNA (45 and references therein), it can be surmised that the altered adenine pairings could represent, in addition to the actual consensus sequence, a general and essential element for driving H-NS to its binding sites.

Many ‘slow’ H-NS protections were found in sequences complementary or adjacent to fast/intermediate H-NS protections. Thus, their presence can be easily explained by the propagation of H-NS on the regions surrounding the nucleation sites. However, protections in regions around −100 and between −270 and −280, are exceptions to this rule and notably, they neither fall in AT-rich regions nor coincide with sequences bound by H-NS*ctd*. Since site I and site II in the *virF* promoter are not entirely independent of each other, as demonstrated by the fact that the lack of the promoter distal site II causes a strong reduction of H-NS protection in site I (14), we cannot rule out the possibility that the slow H-NS protections found in isolated and low affinity sequences could be the result of long-range interactions mediate by H-NS and probably favored by the intrinsic DNA curvature present between the two H-NS-binding sites (14).

## SUPPLEMENTARY DATA

Supplementary Data are available at NAR Online.

SUPPLEMENTARY DATA
