# Supplementary material for: Time-resolved assembly of a nucleoprotein complex between Shigella flexneri virF promoter and its transcriptional repressor H-NS
Source: Nucleic Acids Res. 2014 Nov 11;42(21):13039–50. doi: 10.1093/nar/gku1052 (PMC4245942; doi:10.1093/nar/gku1052)
Supplement: SUPPLEMENTARY DATA [file supp_42_21_13039__index.html]

Time-resolved assembly of a nucleoprotein complex between Shigella flexneri virF promoter and its transcriptional repressor H-NS — Time-resolved assembly of a nucleoprotein complex between Shigella flexneri virF promoter and its transcriptional repressor H-NS — SUPPLEMENTARY DATA 

# Time-resolved assembly of a nucleoprotein complex between *Shigella flexneri virF* promoter and its transcriptional repressor H-NS

## SUPPLEMENTARY DATA

**Files in this Data Supplement:**

- SUPPLEMENTARY DATA
