# Supplementary material for: Time-resolved assembly of a nucleoprotein complex between Shigella flexneri virF promoter and its transcriptional repressor H-NS
Source: Nucleic Acids Res. 2014 Nov 11;42(21):13039–50. doi: 10.1093/nar/gku1052 (PMC4245942; doi:10.1093/nar/gku1052)
Supplement: SUPPLEMENTARY DATA [file supp_gku1052_nar-02239-f-2014-File010.pdf]

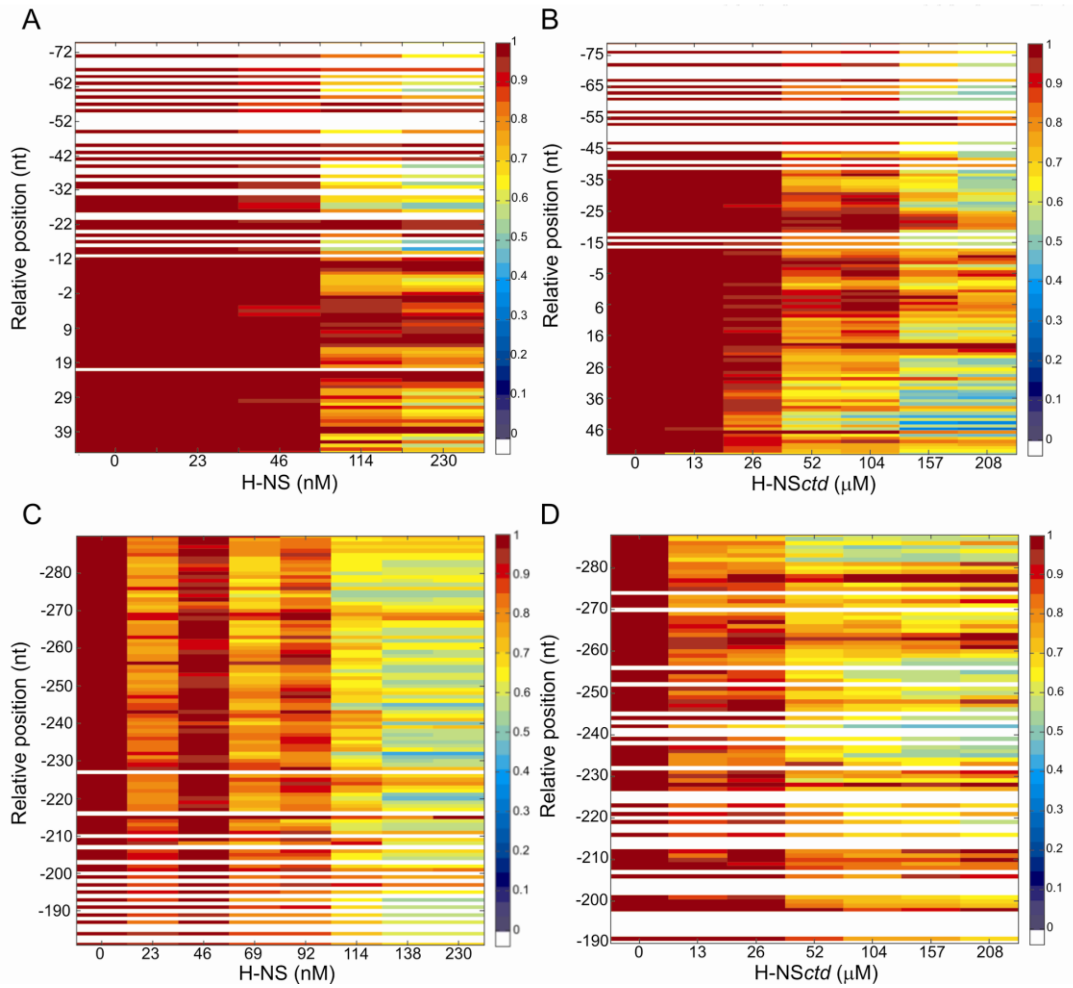

Fig. S1. Quantification of H-NS and H-NS*ctd* protections detected by DNAse I footprinting analysis on the *virF* promoter.
The normalized intensity of the electrophoretically resolved bands of gels shown in Fig. 1 A, B, C and D is represented by boxes of different colours in panels A, B, C and D, respectively. Data were plotted in a linear scale as a function of increasing concentration of H-NS (A and C) and H-NS*ctd* (B and D). A change in colour indicates a change in band intensity due to protein binding, according to the colour key shown in the panels. In brief, dark red corresponds to the intensity measured in the absence of the protein and taken as 100%, red corresponds to a reduction in intensity up to 20%, orange to a reduction between 20%-33%, yellow between 33%-38%, green between 38%-50%, light blue between 50%-63%, blue between 63%-83%, indigo between 80%-100%. White lines indicate absent data or data of poor quality. Numbering is given according to the +1 transcription start site. The intensity of each band was obtained as described in the text and in Material and Methods.
